# COVID-19 impact in radiotherapy practice in an oncology hub: a screenshot from Lombardy, Italy

**DOI:** 10.1177/0300891620980065

**Published:** 2021-12

**Authors:** Giulia Corrao, Luca Bergamaschi, Mattia Zaffaroni, Massimo Sarra Fiore, Giammaria Bufi, Maria Cristina Leonardi, Roberta Lazzari, Daniela Alterio, Federica Cattani, Gabriella Pravettoni, Fabrizio Mastrilli, Roberto Orecchia, Giulia Marvaso, Barbara Alicja Jereczek-Fossa

**Affiliations:** 1 Division of Radiation Oncology, IEO, European Institute of Oncology IRCCS, Milan, Italy; 2 Department of Oncology and Hemato-Oncology, University of Milan, Milan, Italy; 3 Medical Physics Unit, IEO, European Institute of Oncology IRCCS, Milan, Italy; 4 Applied Research Division for Cognitive and Psychological Science, European Institute of Oncology IRCCS, Milan, Italy; 5 Medical Administration, CMO, IEO, European Institute of Oncology IRCCS, Milan, Italy; 6 Scientific Direction, IEO, European Institute of Oncology, IRCCS, Milan, Italy

**Keywords:** Radiotherapy, COVID-19, oncology hub

## Abstract

**Objective:**

During 2020, medical clinical activities were dramatically modified by the coronavirus disease 2019 (COVID-19) emergency. We aim to evaluate the impact of COVID-19 on radiotherapy (RT) practice in a hub cancer center.

**Methods:**

Retrospective data collection of patients with suspected COVID-19 infection, identified by pathognomonic symptoms feedback at triage realized at the entrance to RT division. Inclusion criteria were diagnosis of oncologic disease, COVID-19–related symptoms, and signed written informed consent.

**Results:**

Between 1 March and 30 June 2020, 1,006 patients accessed our RT division for RT simulation or treatment. Forty-four patients matched inclusion criteria (4.4% of all patients): 29 women and 15 men. Seventeen patients had metastatic disease. Twenty-one patients reported fever, 6 presented dyspnea, 4 complained of ageusia and anosmia, and 3 developed conjunctivitis. Thirty-six patients underwent nasal swab, with 7 positive results. From our cohort, 4 cases of pneumonia were diagnosed with computed tomography scan imaging: 3 were related to COVID-19 infection, while the fourth was evaluated as an RT adverse event. From the entire series, 4 patients died: 3 during hospitalization in intensive care unit of complications of COVID-19 and 1 of other causes neither COVID-19 nor cancer-related.

**Conclusions:**

Cancer hub allows for safe RT practice continuation while minimizing the spread of contagion in this frail patient population. A challenge for the future will be to understand pandemic consequences in cancer natural history and manage its clinical impact.

## Introduction

The coronavirus disease 2019 (COVID-19) pandemic has rapidly changed everyday life worldwide.^1^ Lombardy (northern Italy) was the first area in Europe severely hit by the COVID-19 pandemic during the first months of 2020.^2^

Since February 2020, the Italian healthcare system was reorganized to confront the spread of COVID-19 infection and its clinical consequences. In order to ensure better management of the pandemic, several hospitals have been entirely dedicated to COVID-19 treatment.

Due to their fragility and immunosuppression, oncologic patients have been reported to be at higher risk of COVID-19 complications and deaths.^3,4^ During the COVID-19 pandemic, the Regional Health Council converted selected hospitals for cancer care in oncologic hubs to create severe acute respiratory syndrome coronavirus 2 (SARS-CoV-2)–free zones and guarantee the continuation of cancer treatments safely. The European Institute of Oncology (IEO) IRCSS (Milan, Italy) was one of those. COVID-19 infection symptoms are heterogeneous and often difficult to distinguish from cancer manifestations or treatment side effects. In this scenario, a hub hospital should remain COVID-19 free/safe, employing preventive measures and testing every suspect patient with nasopharyngeal swab.

Clinical recommendations for the management of oncologic patients have been provided by many scientific societies, to modify the standard of care and minimize contamination risk, considering the balance between risk and benefits and the need to protect both healthcare professionals and patients.^5^ With approximately half of cancer patients undergoing radiotherapy (RT) during the natural course of their disease,^6^ radiation oncology departments had to reorganize their clinical routine to maintain the same high standard of care of the pre–COVID-19 era.

The current work aims to report our RT division experience as an oncologic hub operating in the first European area hit by the pandemic, with a focus on the management of patients with suspected/verified COVID-19 infection.

## Methods

Data of patients treated at the IEO radiation oncology department from 1 March to 30 June 2020 were retrospectively collected.

Inclusion criteria were the following: any diagnosis of oncologic disease; access to the radiation oncology department; COVID-19–related symptoms reported before, during, and after RT treatment; and signed written informed consent for the use of personal data for scientific and educational purposes.

The study aimed to evaluate the impact of the COVID-19 pandemic on RT practice in a cancer hub and to analyze the efficacy of preventive measures adopted in order to minimize the contagion and to maintain a COVID-19–free/safe hospital.

This study was approved by the ethics committee of Istituto Europeo di Oncologia IEO IRCSS and Centro Cardiologico Monzino IRCSS (notification UID 2349).

## Results

### Radiation oncology department organizational aspects

Approximately 900 accesses every week are registered to our department for RT treatments.

Because RT is administered exclusively on outpatients, a further limitation derives from the impossibility to perform a nasopharyngeal swab screening for all patients, as instead occurs before a scheduled surgical hospitalization.

The entire division workflow was reorganized in order to minimize COVID-19 transmission, rapidly responding to the evolving knowledge about the pandemic.^7^

First, to avoid gatherings in the division, remote working solutions have been implemented for some of the staff (such as data managers and biomedical engineers), while medical doctors and medical physicists have been asked to organize activities in different shifts. Moreover, the multidisciplinary boards have been reorganized as video conference calls.

Second, during the lockdown period, telemedicine was encouraged, providing for a subsequent official report by email, especially in case of follow-up visits or for second opinions.^8^ Clinical private practice was temporarily suspended, according to the regional regulations. Hypofractionated regimens were preferred in order to limit access to the division and nonurgent treatments were momentarily deferred when possible, as suggested in specific guidelines published during the pandemic.^9–13^

Third, admission to the hospital was restricted only to patients, at their established time, without accompanying persons, except in case of clinical need. Waiting rooms for patients are organized with chairs placed at a safe distance between each other.

All patients, visitors, and staff must wear surgical masks. Personal protective equipment (PPE) for staff includes surgical caps, gloves, and protective visors. A double temperature check is performed both at the entrance of the hospital and of the RT division.

IEO provides each scheduled patient with an email form in order to evaluate and possibly isolate those with suspect COVID-19. All inpatients undergo nasopharyngeal swab less than 72 hours before hospitalization.

An emergency team has been created to manage any suspected COVID-19 infection in our division. This team is composed of the department chief, three senior radiation oncologists, the radiation therapist coordinator, the nursing coordinator, and the medical physics unit chief. During the work time, at least one member of the emergency team has to be reachable on a dedicated phone number to address any report promptly.

When patients are admitted to the division for the first time, an informative paper is provided and they are prompted to contact the emergency team number prior to any future access to the division.

In case of suspicious symptoms (fever >37.5°C, cough, dyspnea, anosmia and ageusia, flu syndrome, conjunctivitis, diarrhea), patients are stopped at the entrance of the division and isolated in dedicated areas, pending nasopharyngeal swab, and evaluated for possible transfer to a COVID-19 hospital.

In case of patients reporting COVID-19 symptoms during the treatment in the division, a specific alcohol-based sanitization of all areas is requested.

Since the beginning of the outbreak, specific psychologic support has been offered to patients, their relatives, and staff.

### Patient characteristics

Between 1 March and 30 June 2020, 1,200 patients accessed our RT division for RT simulation or treatment. Forty-four patients matched inclusion criteria (4.4% of all patients), 29 women (66%) and 15 men (34%). The cohort was heterogeneous for sex, age, Karnofsky Performance Status (KPS), tumor site, and TNM staging. Median age was 58.5 (interquartile range 48–68). Most patients (57%) had KPS 100 (25/44), while 17 (39%) had KPS 90 and only 2 (4%) had KPS 80.

Tumor sites were as follows: 34% (15) breast, 21% (9) gynecologic, 18% (8) head and neck, 10% (4) prostate, 7% (3) non-small cell lung cancer, 2% (1) non-Hodgkin lymphoma, 2% (1) neuroendocrine, 2% (1) high-grade sarcoma, 2% (1) rectum, and 2% (1) double site (rectum and breast). Seventeen (39%) patients had metastatic disease. A complete list of baseline characteristics is available in Table 1. Twenty-one (48%) patients reported fever, 6 (14%) presented dyspnea, 4 (9%) complained of ageusia and anosmia, and 3 (7%) developed conjunctivitis. A complete list of symptoms is available in Table 2.

**Table 1.** Baseline characteristics.

Characteristics	N (%) (total n = 44)
Sex	
M	15 (34)
F	29 (66)
Histology	
Breast	15 (34)
Head and neck	8 (19)
Gynecologic	9 (21)
NSCLC	3 (7)
Prostate	4 (9)
LNH	1 (2)
NET	1 (2)
High-grade sarcoma	1 (2)
Rectum	1 (2)
Rectum and breast	1 (2)
TNM stage	
I	6 (14)
II	6 (14)
III	15 (34)
IV	17 (38)
RT setting	
Adjuvant	17 (39)
Palliative	14 (32)
Curative	12 (27)
Oligometastatic	1 (2)
KPS	
100	25 (56)
90	17 (39)
80	2 (5)
Nasopharyngeal swab	
Positive	7 (16)
Negative	29 (66)
Unknown	8 (18)
Last follow-up	
AWD	24 (55)
NED	16 (36)
Died of cancer	0 (0)
Died of COVID-19	3 (7)
Died of other causes	1 (2)

AWD: alive with disease; COVID-19: coronavirus disease 2019; KPS: Karnofsky Performance Status; LNH: non-Hodgkin lymphoma; NED: no evidence of disease; NET: neuroendocrine tumor; NSCLC: non-small cell lung cancer; RT = radiotherapy.

**Table 2.** Patient-reported symptoms.

Symptoms	N (%) (total n = 44)
Fever	21 (48)
Cough	5 (11)
Dyspnea	6 (14)
Flu syndrome	3 (7)
Conjunctivitis	3 (7)
Ageusia/anosmia	4 (9)
Diarrhea	3 (7)
Nausea or vomiting	4 (9)
Pericarditis	1 (2)
Pneumonia	5 (11)
Muscle pain	3 (7)
Abdominal pain	1 (2)
Sore throat	6 (14)
Asthenia	3 (7)

The majority of symptomatic patients (29/44) were identified during the first period of the pandemic (March–April 2020), whereas only 15 patients were located during the second phase, from May to June 2020.

Among the entire cohort, half of the patients experienced RT suspension, with a median time of 7 days (2.75–12.5 interquartile range [IQR]).

Thirty-six patients underwent nasopharyngeal swab, with 7 positive results. Among the latter, 5 were men, median age was 68 (interquartile range 64–77), most had KPS 100 (4), and reported symptoms were dyspnea (4), anosmia and ageusia (3), and flu syndrome (1). Notably, all positive cases were identified during the first pandemic period (March and April).

In this subgroup, 4 patients reported symptoms after the end of RT treatment without any suspension. One interrupted scheduled RT and, after the resolution of the infection, his therapeutic program changed from chemoradiation therapy with a curative intent to surgery followed by adjuvant RT. Two patients died of COVID-19 before starting RT.

Twenty-nine patients underwent nasopharyngeal swab with negative result (80%); among those, 13 (45%) experienced a median RT suspension of 6 days (2–10 IQR).

From our cohort, five cases of pneumonia were diagnosed with computed tomography imaging: three were related to COVID-19 infection, while the remaining were evaluated as an RT adverse event. From the entire series, four patients died, three patients died during hospitalization in intensive care unit for complications of SARS-CoV-2 infection, and one died of causes neither COVID-19 nor cancer-related.

Among patients who died, the median age was 68.5 years (range 58 to 91); three presented metastatic disease, without significant comorbidities in anamnesis. One did not start RT; three of them stopped RT after the third day of treatment.

## Discussion

The COVID-19 pandemic has represented an unprecedented challenge for health care systems. During the first phase of the pandemic, with the aim of reducing the infection spread, the Italian government was the first in Europe to institute a lockdown procedure. Measurements to restrict the spread in public institutions increased gradually and PPE availability shorted in supply in various countries. The reduction of positive cases allowed the beginning of a subsequent phase, starting in May 2020, with the final aim of gradual activities reopening. Simultaneously, increased accessibility of PPE, serologic tests, and nasopharyngeal swabs made it possible to identify affected patients early and maintain adequate safety measures.

One of the most concerning problems during the pandemic peak of COVID-19 infection in Lombardy was the management of oncologic patients, in particular to guarantee proper treatment while minimizing the risk of contagion.^14,15^ Furthermore, the extreme variety of COVID-19 infection symptoms could mimic tumor displays and side effects of RT treatments. Our data confirm that frail patients are at increased risk of life-threatening complications from SARS-CoV-2 infection; all COVID-19–related deaths in our study were among patients with metastatic disease.

During the emergency situation at the beginning of the COVID-19 pandemic, clinical recommendations from national and international oncologic associations (World Health Organization, Italian Association of Radiotherapy and Clinical Oncology [AIRO], European Society for Radiation Oncology [ESTRO], Italian Association of Medical Oncology, European Society of Medical Oncology) helped clinicians cope with the pandemic peak and manage different clinical situations.^12,16–30^

Slotman et al.,^5^ surveyed by ESTRO, presented an international overview of RT patient management during the pandemic, reporting how the radiation oncology community rapidly organized itself to ensure the best treatment options and, at the same time, to protect patients and healthcare professionals.

As shown by a recent survey endorsed by AIRO,^17^ most of the engaged RT departments reported a reduction of their clinical activity between 10% and 30%. In this scenario, according to regional guidelines, due to the high experience in cancer treatments, IEO has become an oncologic hub center, receiving patients from general hospitals converted to COVID-19 units. For this reason, the number of accesses has remained almost unchanged compared to the pre–COVID-19 era. Therefore, it was important to take precautionary measures aimed at protecting patients and staff and maintaining the hub of IEO.

Despite the entity of the pandemic in Lombardy and the high number of daily patient access at our division, the limited number of positive cases in our unit suggests that the measures taken have been adequate. Our data highlight how increasing safety measures are effective to identify patients at risk and to isolate them with a proper diagnostic pathway. In particular, all positive cases were identified during the first phase of pandemic, when PPE was scarcely available or contradictory (recommendations for use of masks) and contagion understanding was weak. With the implementation of checkpoints, sanitation devices, and population awareness campaigns, patients became adequately trained to recognize suspicious symptoms and communicate them to competent physicians. As a result, no COVID-19–positive patients have reached the division since June, allowing us to work safely and protect patients and staff.

Furthermore, the improvement in diagnostic timing permits us to obtain nasopharyngeal swab results on the same day, avoiding RT interruption, unless disabling symptoms such as fever or dyspnea occur.

Our cancer hub series suggests that the choice to send all patients at risk, including cancer patients, to a highly specialized COVID-19–free/safe center has proven to be effective in minimizing the spread of contagion in this frail population. In order to protect oncologic patients, considering the effectiveness of our measures, it will be prudent follow these recommendations until the end of the pandemic. All the preventive measures adopted during the first wave of the pandemic were effective in containing the viral spread, laying the foundation for a more responsible approach in the subsequent phases of the outbreak.

Recent studies highlighted how the pandemic emergency caused a shift from a patient-centered ethic to a public health–centered ethic in order to control the spread.^31–33^ The pandemic promoted the establishment of novel guidelines for the management of oncologic patients. Remote consulting and tele-visits were implemented in order to lower patients’ access to hospitals and for RT wards hypofractionated regimens were preferred. It is likely that some of these changes in clinical routine will be maintained at the end of the emergency, defining a novel patient-centered ethic.^31–33^

A challenge for the future will be to understand the pandemic’s consequences in cancer’s natural history and to better manage its clinical effects.
